# Long-lived adult-born hippocampal neurons promote successful cognitive aging

**DOI:** 10.1038/s41380-025-03286-5

**Published:** 2025-10-01

**Authors:** Nicolas Blin, Vanessa Charrier, Fanny Farrugia, Justine Palhol, Antoine Presset, Estelle Cartier, Stephane Oliet, Emilie Pacary, Muriel Koehl, Dieter Chichung Lie, Nuria Masachs, Djoher Nora Abrous

**Affiliations:** 1 Univ. Bordeaux, INSERM, Magendie, U1215, Neurogenesis and Pathophysiology Group, F-3300 Bordeaux, France; 2 Univ. Bordeaux, INSERM, Magendie, U1215, Glia-neuron interactions Group, F-3300 Bordeaux, France; 3 Institute of Biochemistry, Emil Fischer Center, Friedrich-Alexander Universität Erlangen-Nürnberg, Erlangen, Germany

**Keywords:** Neuroscience, Physiology

## Abstract

Aging is commonly associated with a decline in memory abilities, yet some individuals remain resilient to such changes. Memory processing has been shown to rely on adult neurogenesis, a form of hippocampal plasticity, but whether the integration and role of long-lived adult-born neurons (ABNs) generated during early adult life also contribute to cognitive resilience and to such inter-individual differences remain unknown. Using a pseudo-longitudinal approach in rats characterized as resilient or vulnerable to cognitive aging, we examined the survival, senescence, morphology, glutamatergic connectivity, and mitochondrial health of ABNs. To achieve this, we combined approaches based on thymidine analogues and retroviral labeling using Moloney murine leukemia viruses. While ABNs survival, entry into senescence and dendritic gross morphology did not differ between resilient and vulnerable rats, resilient animals exhibited preserved glutamatergic synaptic input and maintained mitochondrial homeostasis in the proximal dendrites of ABNs. Interestingly, bypassing this reduction in glutamatergic inputs in vulnerable rats through direct optogenetic stimulation was sufficient to rescue their memory retrieval abilities, indicating that ABNs themselves remain intrinsically functional despite reduced input. Overall, our data indicate that maintaining long-lived ABNs within the neuronal network is essential for successful cognitive aging, highlighting their potential as a therapeutic target for restoring cognitive functions in old age.

## Introduction

Cognitive aging has emerged as a global challenge due to the growing elderly population [1]. Cognitive aging is associated with a decline in memory functions, particularly episodic memory which involves recalling personal experiences. Interestingly, this decline varies among individuals, with some remaining resilient with preserved memory functions while others are vulnerable with decreased abilities [2–5]. These inter-individual differences have also been described in rodents [2, 3] especially in tasks measuring spatial memory abilities, making them a valuable model for studying this issue.

Memory processes depend on the creation of new neurons in the dentate gyrus of the hippocampus [6, 7]. Aging is associated with an exhaustion of the pool of new neurons and their delayed maturation [8–10]. Interestingly, increasing this pool at old age was shown to improve memory abilities [11–14]. However, it is unclear whether neurons born earlier in adult life are also involved in the aging of cognitive functions since most studies focused on neurons produced at old age only. The few data available indicate that these neurons are long-lived as they survive for years and express markers of neuronal activity in response to learning [15, 16].

We tested whether successful aging relies on preserving the health and proper neuronal network integration of mature and long-lived adult-born neurons (ABNs), allowing for accurate reception of incoming information and subsequent recruitment. We observed that ABNs in resilient animals display preserved glutamatergic innervation in their proximal dendrites, which typically receive inputs from mossy cells and the supra-mammillary area (SuM) of the hypothalamus, suggesting that these inputs may be selectively affected by aging. In contrast, ABNs in vulnerable animals experience a disconnection from this network. Additionally, these proximal dendritic synaptic sites in ABNs demonstrate a maintenance of their mitochondrial homeostasis. Despite these alterations, bypassing the loss of inputs with optogenetic stimulation successfully restored memory abilities in old vulnerable animals. Altogether, ABNs represent a valuable tool for restoring memory abilities in old age.

## Materials and methods

### Rats

A total number of 294 male Sprague-Dawley rats (OFA, Janvier, France) were used for these experiments. Rats aged 3-month-old at the time of delivery were group-housed in standard cages under a 12/12 h light/dark cycle with ad libitum access to food and water. Temperature (22 °C) and humidity (60%) were kept constant. Rats were individually housed before the beginning of training at either the end of adulthood (8-month-old), middle-age (12-month-old) or old age (18 or 20-month-old). Experimental procedures were carried out following the European directive of the parliament and the Council of September 22, 2010 (2010/63/UE). Animal studies were approved by the ethical committee of the University of Bordeaux (ID #10864, #11155, #21501, #5012006 A).

### Thymidine analog injections

Newly born cells were labeled by the incorporation of the synthetic thymidine analog BrdU (5-bromo-2′-deoxyuridine) (Sigma, Cat#B5002) dissolved in a Phosphate Buffer (pH 8.4). Rats received four injections (50 mg/kg/day) intra-peritoneally during four consecutive days when 3-month-old.

### Retroviruses

The M-rv-GFP (gift from Prof. F.H. Gage, Salk Institute, La Jolla, CA) enables the visualization and morphological reconstruction of infected neurons for structural analysis, as previously described [17]. The M-rv-PSD95-GFP (gift from Dr. C. Lois, Caltech, Pasadena, CA) labels the postsynaptic density of dendritic spines, allowing for the quantification of glutamatergic innervation [18]. The M-rv-MitoDsRed (gift from Dr. D.C. Lie, Friedrich-Alexander Universität Erlangen-Nürnberg, Germany) selectively labels mitochondria within infected neurons, enabling the quantification of the density in mitochondrial clusters [19]. The M-rv-Channelrhodopsin-GFP (gift from Dr. S. Ge, Stony Brook University, New York, NY) allows the expression of a light-sensitive receptor at the neuronal membrane for optogenetic stimulation of infected neurons [20]. High retroviruses titers (between 5 × 10^8^ TU/mL and 5 × 10^9^ TU/mL) were prepared with a human 293-derived retroviral packaging cell line (293GPG) kindly provided by Dr D.C. Lie. Virus-containing supernatant was harvested three days after transfection with Lipofectamine 2000 (Thermofisher). This supernatant was then cleared from cell debris by centrifugation at 3500 rpm for 15 min and filtered through a 0.45 μm filter (Millipore). Viruses were concentrated by two rounds of centrifugation (19 500 rpm 2 h) and resuspended in PBS.

### Surgery

Rats were anaesthetized with 3% isoflurane and placed in the stereotaxic frame, where they were maintained asleep with 2% isoflurane for the duration of the surgery. Analgesia was provided via injections of Metacam (1 ml/kg, sub-cutaneous, Boehringer Ingelheim) and lidocaine (0.1 ml, sub-cutaneous, Vetoquinol) at the incision site.

### Retroviral injections

A retroviral mix of half M-rv-GFP and half M-rv-MitoDsRed was prepared before injection. The retroviral injections (2 μL per injection site at 0.3 μL/min) were stereotaxically made into the dentate gyrus of 3-month-old adult rats with a microcapillary pipette connected to a Hamilton syringe placed into a micro injector (KDS legato 130) directly attached to the stereotaxic frame. Cannulas were kept in place for 3 min after the end of the injections to allow full diffusion of the viral suspension. The Moloney leukemia virus-based retroviral vector M-rv-PSD95-GFP and mixed M-rv-GFP/M-rv-MitosDsRed were injected to the right and left dorsal dentate gyrus respectively. For the optogenetic experimental cohorts, the M-rv-Channelrhodopsin-GFP was injected bilaterally to the dorsal area of the dentate gyrus. Injections were administered using the following coordinates: [AP]:−3,5 mm; [L]:−/ + 1,7 mm; [P] from skull:−4,3 mm.

### Optic fiber implantations

Home-made optic fibers were made following a protocol previously described [21]. The implantation surgical procedure was carried out one month before the start of behavior, following the same protocol as that described for the retroviral injection. The skull was scratched and three anchor screws were fixed (one in the anterior area and two in the posterior area). The two optic fibers [length: 4.5 mm, diameter: 200 μm, numeric aperture (NA 0.37)] were lowered with a speed of ~2 mm/min upwards to the M-rv injection site with the following stereotaxic coordinates: [AP]:−4.1 mm; [L]:−/ + 1.8 mm; [P] from *dura-mater*:-3mm. Fibers were stabilized using dental cement. When the cement was dry, scalps were sutured and disinfected with local antiseptic treatment (Betadine). All rats were followed up until the start of the behavior. During handling, fur was checked and the scar disinfected systematically. Rats were handled every day to habituate them before the behavioral procedure.

### Watermaze procedures

The apparatus consisted of a circular plastic swimming pool (180 cm diameter, 60 cm height) filled with water (20 ± 1 °C) rendered opaque by the addition of a white cosmetic adjuvant. Two days before training, the animals were habituated to the pool for 1 min. For old animals, we followed previous protocol published for aging experiments by our laboratory [22]. The capacity to swim and to see correctly were assessed during a training phase where animals had to locate a visible platform within the swimming pool, all rats with deficits were removed from the experiments (i.e. rats that did not find the platform in less than 20 s after 5 day of platform training). During training, animals were required to locate a submerged platform hidden 1.5 cm under the surface of the water in a fixed location, using the spatial cues available in the room. Rats were trained for four trials per day (90 s with an inter-trial interval of 30 s and released from three different starting points that varied randomly each day). If an animal failed to locate the platform, it was placed on the platform at the end of the trial for the duration of the inter-trial interval. The distance crossed to reach the platform was recorded using a video camera that was secured to the ceiling of the room and connected to a computerized tracking system (Videotrack, Viewpoint). Daily results were analyzed in order to rank animals according to their behavioral score calculated over the last 3 days of training. Training stopped before the resilient animals reached plateau phase of learning.

### Resilient and vulnerable extremes populations for morphological analyses

Following the end of learning, animals were cognitively attributed according to the mean performances of their three last days of training. From the whole population undergoing the learning task, the five animals with the highest score (i.e. shortest mean distance to reach the platform) were attributed as extreme resilient and the five animals with the lowest score (longest mean distance to reach the platform) were attributed as extreme vulnerable, replicated in three independent aged-cohorts. Only the extremes were kept for analysis as considered the most representative of their cognitive abilities, as already published by our laboratory [22].

### Stability of the behavioral phenotype

To assess whether the cognitive status of individual animals remained stable over aging, an additional cohort was subjected to the watermaze task at both 8 and 18 months of age (Figure S2). Correlation analysis was performed using the mean performance across the last three training days for each learning training at both ages.

### Watermaze for optogenetic manipulation of ABNs

The watermaze was equipped with a blue laser source (OptoDuet 480 nm, 200 mW, Ikecool) connected to a rotatory joint to avoid tangling of the patch cords connected to the intra-dentate gyrus optic fibers. Light intensity at the optic fiber cable tip was controlled every day before the beginning of the session (8–10 mV). Illumination was applied throughout the session at 20 Hz pulses of 15 ms. The time to reach the platform was recorded using a video camera connected to a computerized tracking system (Polyfiles, Imetronic). Light stimulation was provided during the 4 trials each day for the entire learning phase. When resilient animals reached the plateau phase, training was stopped and 48 h later animals were tested during a probe test for 60 s where no animals without light stimulation. During the probe test, the hidden platform was removed and the time spent in each quadrant was quantified. Control animals consisted of animals that underwent the same procedure but without light stimulation. Resilient and vulnerable populations were selected from the stimulated and non-stimulated groups respectively using the mean performance across the last three training days for each animal. Optogenetic experiments were replicated at 12–18-month-old.

### Immunohistochemistry and analysis

For animals injected with M-rv-GFP, M-rv-MitoDsRed, M-rv-PSD95-GFP and M-rv-Channelrhodospin-GFP, animals were perfused transcardially with a phosphate-buffered solution of 4% paraformaldehyde 90 min after the last training session. After 24 h fixation, optogenetic brains were cut using a vibratome. For PSD95-GFP and mitoDsRed, after 24 h of fixation, brains were transferred in a PBS-sucrose (30% solution for 24 h and then cut using a vibratome.

### BrdU and senescence-associated-ß-galactosidase (SA-ß-Gal) positive cells

Free-floating 50-μm-thick sections were processed according to a standard immunohistochemical procedure on alternate one-in-ten sections to visualize BrdU^+^ cells throughout the entire granular layer of the dentate gyrus with an anti-BrdU primary antibody (Mouse, 1:50, SantaCruz, Cat#sc-32323). Bound antibodies were visualized using the biotin-streptavidin technique (ABC kit, Vector Laboratories Inc., Cat#PK-4000) and 3,3′-diaminobenzidine as a chromogen with a biotinylated horse anti-mouse antibody (1:200, Vector Labs, Peterborough, UK. Cat#BA-2001). Primary antibody was incubated at 4 °C for 72 h, and secondary antibody was incubated at room temperature (RT) for 2 h.

SA-ß-Gal^+^ cells were revealed using the Senescence β-Galactosidase Staining Kit (Cell Signaling, #9860) according to the manufacturer’s instructions. The number of BrdU^+^ cells and SA-ß-Gal^+^ cells in the dentate gyrus was estimated with systematic random sampling of every tenth section along the septo-temporal axis of the hippocampal formation using a modified version of the optical fractionator method. All of the BrdU-SA-ß-Gal^+^ cells were counted on each section and the resulting numbers were tallied and multiplied by the inverse of the section sampling fraction (1/ssf = 10). The percentage of colocalization BrdU-SA-ß-Gal^+^ cells was calculated as follows: (Nb of BrdU^+^-SA-ß-Gal^+^) / (Nb of BrdU^+^-SA-ß-Gal^+^ + Nb of BrdU^+^-SA-ß-Gal^-^) x 100. All analyses were conducted by an experimenter blind to group assignment.

### GFP expressing cells

Free-floating 50-μm-thick sections were processed according to a standard immunohistochemical procedure on alternate one-in-ten sections to visualize the retrovirus-expressing GFP with an anti-GFP primary antibody (Rabbit, 1:12000; Millipore, Cat#AB3080P). Bound antibodies were visualized using the biotin-streptavidin technique (ABC kit, Vector Laboratories Inc., Cat#PK-4000) and 3,3′-diaminobenzidine as a chromogen with a biotinylated goat anti-rabbit antibody (1:200, Vector Laboratories Inc., Cat#BA-1000). Primary antibody was incubated at 4 °C for 72 h, and secondary antibody was incubated at room temperature (RT) for 2 h. The morphometric analysis of virus-labelled neurons was performed with a × 100 objective. Measurements of dendritic parameters as well as Sholl analysis were performed with the Neurolucida software (Microbrightfield, Colchester, VT, USA). The analysis of the dendritic arborization (low and high order repartition, branching angle and path distance calculations) was performed as previously described [23]. Only animals with at least 4 neurons who could be reconstructed and neurons with a minimum of 4 ramifications point were kept for analysis. All analyses were conducted by an experimenter blind to group assignment.

### PSD95-GFP and mitoDsRed expressing cells

Free-floating 50-μm-thick sections were processed according to standard immunohistofluorescence procedure on alternate one-in-ten sections to visualize the retrovirus-expressing eGFP and mitoDsRed with an anti-GFP primary antibody (Rabbit, 1:500, Millipore, Cat#AB3080P) and anti-DsRed primary antibody (Rabbit, 1:1000, Clontech Takara, Cat#632496). Bound antibodies were visualized with Cy3 goat anti-rabbit (Jackson Immuno Research, 1:1000, Cat#111-167-003) secondary antibody. Primary antibodies were incubated simultaneously at 4 °C for 72 h, and secondary antibodies were incubated simultaneously at room temperature (RT) for 2 h. Fluorescence was studied using a SPE confocal system with a plane apochromatic X 63 oil lens (numerical aperture 1.4; Leica) and a digital zoom of 2,5. For both approaches, MosaicJ plugin from ImageJ was used to reconstruct the neuron. Mosaic reconstruction was used to determine the length of the molecular layer and to delimitate the inner, middle and outer sub-layers of the molecular layer. For each layer, dendritic segments of minimum 15 µm and maximum 50 µm were analyzed (minimum of 3 neurons per animals, 5 animals per group). The number of clusters of PSD95-GFP and mitoDsRed was determined either manually or semi-automatically using an intensity threshold in Image J. The density of clusters was obtained by dividing the number of clusters by the length of the corresponding dendritic segment. All analyses were conducted by an experimenter blind to group assignment.

### vGLUT2 labelling

Free-floating 50-μm-thick sections were processed according to standard immunohistofluorescence procedure on alternate one-in-ten sections to visualize vGLUT2 in pre-synaptic terminals with an anti-vGLUT2 primary antibody (Rabbit, 1:250; Synaptic Systems, Cat#135403). Bound antibodies were visualized with Cy3 goat anti-rabbit (Jackson Immuno Research, 1:1000, Cat#111-167-003) secondary antibody. Primary antibody was incubated at 4 °C for 72 h, and secondary antibody was incubated at room temperature (RT) for 2h30. Fluorescence was studied using a SPE confocal system with a plane apochromatic X 20 oil lens (numerical aperture 1.4; Leica) and a digital zoom of 1. Acquisitions were performed in the dorsal dentate gyrus followed by the reconstruction of the acquired dentate gyrus using mosaics, resulting in 3 mosaic-reconstructed dentate gyri analyzed per animal. An Image J macro was used to delimitate the inner, middle and outer sub-layers of the molecular layer on the mosaic and to calculate the mean intensity of vGLUT2 fluorescent labelling. The mean intensity for vGLUT2 labelling was obtained by dividing the mean intensity of each area analyzed (mean intensity / µm2). All analyses were conducted by an experimenter blind to group assignment.

### Mossy cells GluR2/3 labelling

Free-floating 50-μm-thick sections were processed according to a standard immunohistochemical procedure on alternate one-in-ten sections to visualize GluR2/3 labelling expressed in mossy cells with an anti-GluR2/3 primary antibody (Rabbit, 1:50; Millipore, Cat#AB1506). Primary antibody was incubated at 4 °C for 72 h, and secondary antibody was incubated at room temperature (RT) for 2 h. Bound antibodies were visualized using the biotin-streptavidin technique (ABC kit, Vector Laboratories Inc., Cat#PK-4000) and 3,3′-diaminobenzidine as a chromogen with a biotinylated goat anti-rabbit antibody (1:200, Vector Laboratories Inc., Cat#BA-1000). The total number of mossy cells were counted under a X 40 microscope objective all along the temporal–septal axis of the left and right dentate gyrus. The total number of cells was estimated using the optical fractionator method, and the resulting numbers were tallied and multiplied by the inverse of the sections sampling fraction (1/ssf = 10). All analyses were conducted by an experimenter blind to group assignment.

### Channelrhodospine-GFP expressing cells

Free-floating 50-μm-thick sections were processed according to a standard immunohistochemical procedure on alternate one-in-ten sections to visualize the retrovirus-expressing GFP with an anti-GFP primary antibody (Rabbit, 1:2000; Millipore, Cat#AB3080P). Primary antibody was incubated at 4 °C for 72 h, and secondary antibody was incubated at room temperature (RT) for 2 h. Bound antibodies were visualized using the biotin-streptavidin technique (ABC kit, Vector Laboratories Inc., Cat#PK-4000) and 3,3′-diaminobenzidine as a chromogen with a biotinylated goat anti-rabbit antibody (1:200, Vector Laboratories Inc., Cat#BA-1000). Channelrhodopsin-GFP^+^ cells were counted under a × 100 microscope objective throughout the entire septotemporal axis of the granule layer of the dentate gyrus. The total number of cells was estimated using the optical fractionator method, and the resulting numbers were tallied and multiplied by the inverse of the sections sampling fraction (1/ssf = 10). All analyses were conducted by an experimenter blind to group assignment.

### Channelrhodospine-GFP and Zif268 colocalisation

The activation of Channelrhodopsine-GFP^+^ cells was studied using immunohistofluorescence procedure on alternate one-in-ten sections. The retrovirus-expressing GFP was visualized with an anti-GFP primary antibody (Chicken-1:1000, Abcam, Cat#ab13970) and cellular activity with an anti-Zif268 antibody (Rabbit-1:100, Santa Cruz Biotechnology, Cat#sc-189). Bound antibodies were visualized with Alexa488 goat anti-chicken (Thermo Fisher, 1:1000, Cat#A-11039) and Cy3 goat anti-rabbit (Jackson Immuno Research, 1:1000, Cat#111-167-003) secondary antibodies. Primary antibodies were incubated simultaneously at 4 °C for 72 h, and secondary antibodies were incubated simultaneously at RT for 2 h. Fluorescence was studied using a SPE confocal system with a plane apochromatic X 63 oil lens (numerical aperture 1.4; Leica) and a digital zoom of 2,5. The percentage of GFP cells expressing immediate early gene (IEG) (all along the temporal–septal axis) was calculated as follows: (Nb of GFP^+^-IEG^+^ cells)/(Nb of GFP^+^-IEG^− s^cells + Nb of GFP^+^-IEG^+^ cells) × 100. In middle-age experiments, every GFP^+^ were analyzed in the left and right dentate gyrus. In old age experiments, every GFP^+^ cells were analyzed in the left dentate gyrus. All analyses were conducted by an experimenter blind to group assignment.

### Statistical analysis

All analyses were carried out using the software GraphPad Prisms 8. Data (mean ± SEM) were analyzed using the Student *t*-test (two-tailed, paired or unpaired when appropriate) or one-way, three-way and repeated-measures two-way ANOVA, then followed by Tukey’s comparison test when necessary. Data were tested for normality. Statistical significance *P ≤ 0.05, **P < 0.01, ***P < 0.001.

## Results

### Strategy to follow the aging of ABNs and memory abilities

To analyze the lifelong survival of ABNs born in young adult rats (3-month-old), animals were injected with thymidine analog (Bromodeoxyuridine (BrdU), intraperitoneal injections) which is incorporated into dividing cells at the time of injection. To specifically study the morphological features of ABNs, Moloney leukemia virus-based retroviral vectors (M-rv) were injected into the dentate gyrus as M-rv infect dividing cells at the time of the injections and shortly after [17]. Different M-rv were used to study the morphology (M-rv-GFP [17]; which allows reconstruction of the morphology of infected neurons), glutamatergic post-synaptic density (M-rv-PSD95-GFP [18]; allowing labeling of the scaffolding protein in post-synaptic spines in which glutamatergic inputs are impinging) and mitochondrial network (M-rv-MitoDsRed [19], allowing to labels the clusters of mitochondria in infected neurons) of ABNs.

To examine the progressive mechanisms underlying successful aging, we adopted a pseudo-longitudinal strategy in which rats underwent a spatial navigation learning task in the Morris watermaze either at the end of adulthood (8-month-old rats), middle-age (12-month-old rats) or old age (18-month-old rats). This task was chosen as the gold standard method to study cognitive aging as it depends upon adult neurogenesis [24] and is highly sensitive to aging [3].

Within each of the three cohorts of rats (adult, middle-aged, old), resilient and vulnerable animals were identified. Subsequently, the morphological analysis was performed in rats with the best memory abilities (n = 5, Figure S1), and rats with the worst memory abilities (n = 5, Figure S1). We then verified that the classified resilient and vulnerable rats have similar performance between each methodology or age. A three-way ANOVA revealed significant effects of age (F2,96 = 13.83, P < 0.001) and cognitive status (F1,96 = 326.55, P < 0.001), but no effect of methodology (F3,96 = 2.339, P > 0.05) or age x cognitive status x methodology interaction (F6,96 = 0.466, P > 0.05), confirming the consistency of resilient and vulnerable classification. Additionally, we confirmed in an independent cohort that cognitive status remained stable across aging. Specifically, learning performance at 8 months strongly correlated with performance at 18 months in the same animals (Figure S2).

### Cell survival and senescence of ABNs do not account for successful cognitive aging

**Fig. 1:**
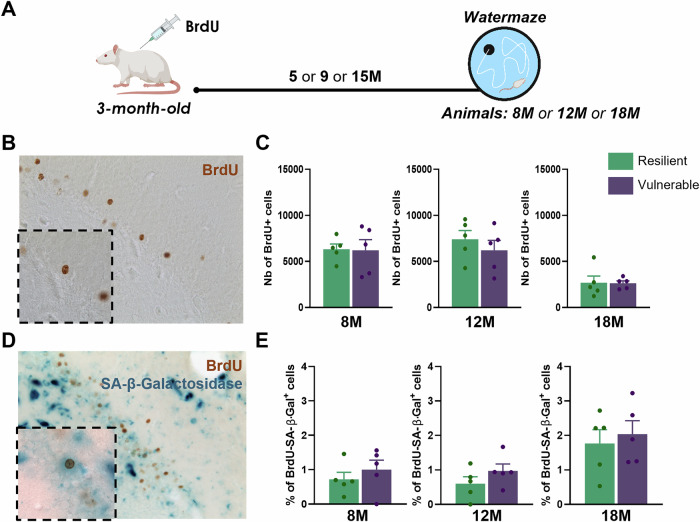
Resilience to cognitive aging is not associated with ABNs death or entry into cellular senescence. **A** Schematic diagram of the experimental design. **B** Picture of a labelled ABN (Brdu^+^). **C** Resilience to cognitive aging is not linked to different survival of ABNs at 8-month-old (unpaired *t* test: *t*_*8*_ = 0.09, P > 0.05), 12-month-old (unpaired *t* test: *t*_*8*_ = 0.83, P > 0.05) and 18-month-old (unpaired *t* test: *t*_*8*_ = 0.94, P > 0.05). **D** Picture of a senescent ABN (Brdu^+^-SA-β-Gal^+^). **E** Resilience to cognitive aging is not linked to higher levels of senescent ABNs at 8-month-old (unpaired *t* test: *t*_*8*_ = 0.81, P > 0.05); 12-month-old (unpaired *t* test: *t*_*8*_ = 1.29, P > 0.05) or 18-month-old (unpaired *t* test: *t*_*8*_ = 0.48, P > 0.05). Data are presented as mean ± S.E.M. from 5 extreme animals per group. Statistical significance *P ≤ 0.05, **P < 0.01, ***P < 0.001.

We first examined whether the survival of ABNs tagged in young rats with BrdU was similar between resilient and vulnerable animals in the three cohorts of different ages cognitively characterized in the watermaze (Fig. 1A, B and Figure S1A), as it could contribute to memory deficits. At all time-points, the total number of BrdU labeled cells was similar between resilient and vulnerable animals (Fig. 1C).

Because cellular senescence corresponds to functional cellular arrest and has been described in aged stem cells [25] and in post-mitotic cells [26], we used the Senescence-Associated-ß-Galactosidase (SA-ß-Gal) marker to detect entry into senescence of ABNs [27], as it may account for their survival but reduced recruitment in vulnerable individuals with age [16]. Senescent ABNs were detected and quantified with visualization of cells co-expressing BrdU and SA-ß-Gal (Fig. 1D). Resilient and vulnerable animals showed similar levels of senescent ABNs for all ages (Fig. 1E). The same profile was observed for the total number of senescent cells (SA-ß-Gal positive) in the granule cell layer of the dentate gyrus (Figure S3). Therefore, while ABNs do not die over the period of 1–2 years, their entry into cellular senescence does not explain the cognitive differences between resilient and vulnerable animals.

### The gross morphological architecture of ABNs is independent of the cognitive status of the rats

**Fig. 2:**
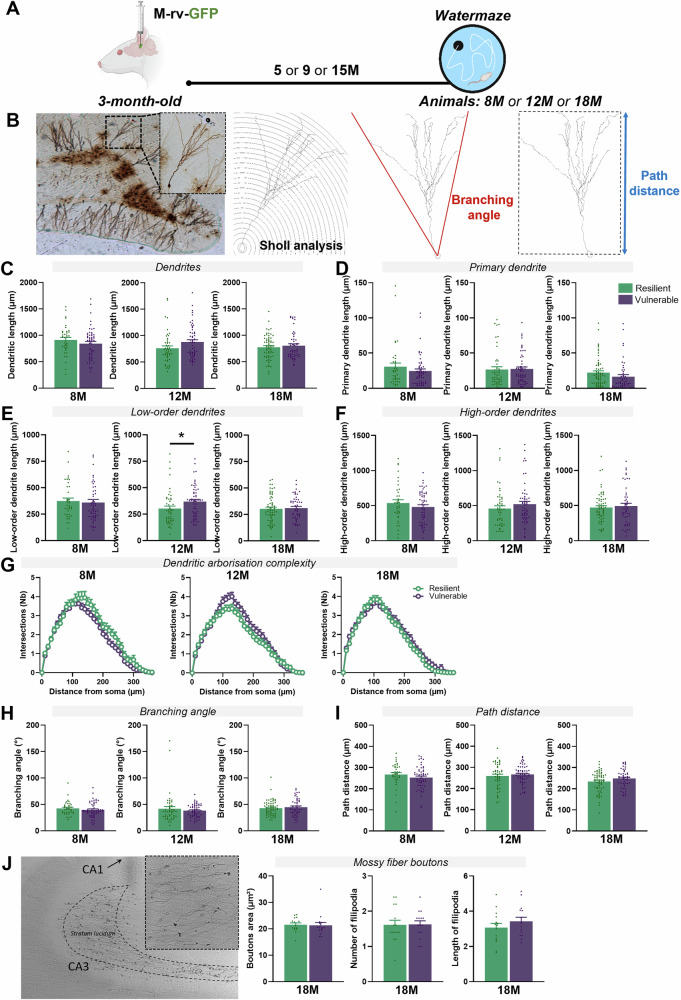
The gross morphological architecture of ABNs is independent of the cognitive status of the rats. **A** Schematic diagram of the experimental design. **B** Picture of a labelled ABN and examples of Sholl analysis for dendritic complexity, branching angle and path distance calculations. **C** ABNs in the two cognitive populations show similar total dendritic length at 8-month-old (unpaired *t* test: *t*_*90*_ = 1.15, P > 0.05), 12-month-old (unpaired *t* test: *t*_*102*_ = 1.98, P > 0.05) and 18-month-old (unpaired *t* test: *t*_*111*_ = 0.61, P > 0.05). **D** ABNs have similar primary dendritic length in the two cognitive populations at 8-month-old (unpaired *t* test: *t*_*90*_ = 1.11, P > 0.05), 12-month-old (unpaired *t* test: *t*_*102*_ = 0.15, P > 0.05), and 18-month-old (unpaired *t* test: *t*_*111*_ = 1.50, P > 0.05). **E** ABNs have similar low-order dendrites length in the two cognitive populations at 8-month-old (unpaired *t* test: *t*_*90*_ = 0.34, P > 0.05) and 18-month-old (unpaired *t* test: *t*_*111*_ = 0.76, P > 0.05). A small increase in vulnerable animals could be observed at 12-month-old (unpaired *t* test: *t*_*102*_ = 2.12, P ≤ 0.05). **F** ABNs show similar high-order dendrites length between the two cognitive populations at 8-month-old (unpaired *t* test: *t*_*90*_ = 1.11, P > 0.05), 12-month-old (unpaired *t* test: *t*_*102*_ = 0.15, P > 0.05) and 18-month-old (unpaired *t* test: *t*_*111*_ = 1.50, P > 0.05). **G** ABNs of resilient and vulnerable individuals have similar dendritic complexity at 8-month-old (RM-two-way ANOVA, F_36,3240_ = 1.09, P > 0.05), 12-month-old (RM-two-way ANOVA, F_36,3408_ = 1.93, P < 0.001) and 18-month-old (RM-two-way ANOVA, F_36,3996_ = 0.84, P > 0.05). **H** ABNs in the two cognitive populations show similar branching angles at 8-month-old (unpaired *t* test: *t*_*90*_ = 0.99, P > 0.05), 12-month-old (unpaired *t* test: *t*_*102*_ = 0.87, P > 0.05) and 18-month-old (unpaired *t* test: *t*_*111*_ = 0.64, P > 0.05). **I** ABNs show similar path distance between the two cognitive populations at 8-month-old (unpaired *t* test: *t*_*90*_ = 1.30, P > 0.05), 12-month-old (unpaired *t* test: *t*_*102*_ = 0.69, P > 0.05) and 18-month-old (unpaired *t* test: *t*_*111*_ = 1.63, P > 0.05). **J** ABNs show similar MFBs area (unpaired *t* test: *t*_*28*_ = 0.17, P > 0.05), number (unpaired *t* test: *t*_*28*_ = 0.08, P > 0.05) and length (unpaired *t* test: *t*_*28*_ = 1.10, P > 0.05) of filipodia between resilient and vulnerable animals at 18-month-old. Data are presented as mean ± S.E.M. from 5 extreme animal per group (a min of 4 neurons were traced per animal, with 8M-Res = 37 neurons and 8M-Vul = 55 neurons; 12M-Res = 48 neurons and 12M-Vul = 58 neurons; 18M-Res = 63 neurons and 18M-Vul = 50 neurons. Statistical significance *P ≤ 0.05, **P < 0.01, ***P < 0.001.

One of the hallmarks of neuronal aging is an atrophy of the dendritic network associated with a loss of inputs [28]. To specifically study the morphological features of ABNs, M-rv coupled with GFP (M-rv-GFP [17]) were injected in the dentate gyrus of 3-month-old rats. These M-rv infect ABNs born at the time of the surgery [17] and allow to label and analyze their morphology (Fig. 2A, B). Three cohorts of rats of different ages were cognitively characterized (Figure S1B), and the total dendritic length of ABNs (Fig. 2C), their primary dendrite length (Fig. 2D), number of nodes (Figure S4A) and numbers of ends (Figure S4B) were shown to remain similar and independent of the cognitive status of the animals.

To better investigate the dendritic complexity, we analyzed the dendrites of ABNs according to their branching order, shown as a good example for sub-dendrite complexity in dentate neurons [23]. However, the ABNs of resilient and vulnerable rats still displayed similar length and number of low and high-order dendrites (Fig. 2E, F and Figure S4C-S4D). Moreover, Sholl analysis revealed that their dendritic arbor had similar complexity (Fig. 2G and Figure S4E). In order to further investigate the ABNs integration within the dentate gyrus, the branching angle and path distance of the dendrites were calculated. ABNs in resilient and vulnerable rats also displayed similar integration for these parameters (Fig. 2H, I).

Given this apparent unchanged dendritic morphology, we investigated the morphology of the ABNs outputs at the level of their mossy fiber boutons (MFBs) in the CA3 at the oldest time point (rats aged of 18-month-old). No differences in the MFBs area nor in the number and length of filipodia could be observed, suggesting preserved synaptic outputs in both resilient and vulnerable rats (Fig. 2J). Additionally, ABNs in resilient and vulnerable rats showed similar cell body morphologies (Figure S4F). Altogether, no major changes in the gross morphology of ABNs could be evidenced between resilient and vulnerable rats in the course of aging.

### Glutamatergic inputs onto the proximal dendrites of ABNs are maintained in resilient rats

Since no major change in the gross morphology of ABNs could be evidenced in the course of aging, we refined our analysis by studying possible connectivity alterations that may lead to altered integration of ABNs within the neuronal network. We focused on the glutamatergic inputs of ABNs, as they represent the main excitatory source essential for neuronal activity and, as such, play a key role in spatial learning.

**Fig. 3:**
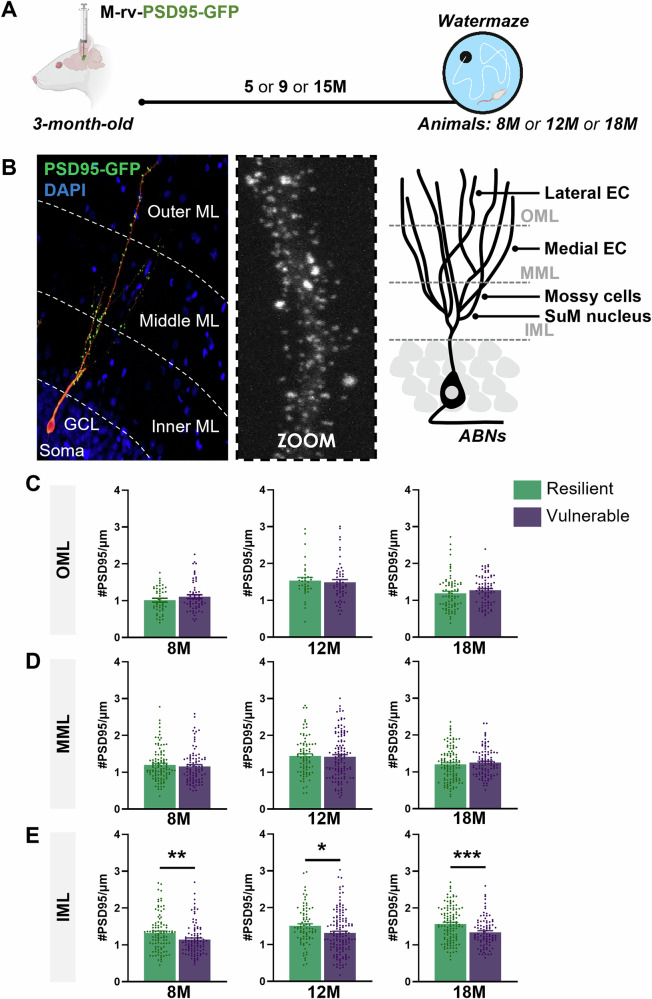
Glutamatergic inputs onto the proximal dendrites of ABNs are maintained in resilient rats. **A** Schematic diagram of the experimental design. **B** Picture of a labelled ABN and the subsequent molecular layer (ML) division in three sub-layers; inner (IML), middle (MML) and outer (OML) molecular layer with their different glutamatergic inputs. **C** Resilience to cognitive aging is not linked to the preservation of glutamatergic inputs in the OML at 8-month-old (unpaired *t* test: *t*_104_ = 1.27, P > 0.05), 12-month-old (unpaired *t* test: *t*_*87*_ = 0.38, P > 0.05) or 18-month-old (unpaired *t* test: *t*_154_ = 1.20, P > 0.05). **D** Resilience to cognitive aging is not linked to the preservation of glutamatergic inputs in the MML at 8-month-old (unpaired *t* test: *t*_*193*_ = 0.65, P > 0.05), 12-month-old (unpaired *t* test: *t*_*199*_ = 0.29, P > 0.05) or 18-month-old (unpaired *t* test: *t*_*210*_ = 0.83, P > 0.05). **E** Resilience to cognitive aging is related to the preservation of glutamatergic inputs in the IML at 8-month-old (unpaired *t* test: *t*_*195*_ = 2.73, P < 0.01), 12-month-old (unpaired *t* test: *t*_*220*_ = 2.51, P ≤ 0.05) and 18-month-old (unpaired *t* test: *t*_*206*_ = 3.62, P < 0.001). Data are presented as mean ± S.E.M. from 5 extreme animals per group (a min of 3 neurons were analyzed per animal, with 8M-Res = 27 neurons and 8M-Vul = 27 neurons; 12M-Res = 21 neurons and 12M-Vul = 33 neurons; 18M-Res = 30 neurons and 18M-Vul = 29 neurons. Statistical significance *P ≤ 0.05, **P < 0.01, ***P < 0.001.

Glutamatergic inputs project to dendritic spines, which are characterized by the presence of the post-synaptic density (PSD), where the PSD95 scaffolding protein is present in high quantity [29]. We used PSD95 as a proxy for glutamatergic inputs impinging onto the spines of ABNs by injecting a M-rv-PSD95-GFP expressing PSD95 coupled with eGFP [18] (Fig. 3A). Since the dentate gyrus receives specific and identified inputs from different glutamatergic regions in each of the sub-layers of the ML [outer molecular layer (OML), middle molecular layer (MML) and inner molecular layer (IML)] (Fig. 3B), the quantification of clusters of PSD95 was made for each sub-layer.

For all aged-population cognitively assessed (Figure S1C), ABNs in vulnerable individuals exhibited a significant reduction in post-synaptic densities specifically in the IML, while they remained comparable between groups in the OML and MML (Fig. 3C-E). This selective loss of synaptic input in the IML of vulnerable animals suggests that inputs to the proximal dendrites of ABNs are particularly affected in cognitive aging. In contrast, resilient animals showed a preservation of PSD95 density in the IML across all ages (Fig. 3E), indicating that the maintenance of proximal synaptic inputs may contribute to their preserved cognitive function.

To verify that our results were specific to ABNs, we quantified the total glutamatergic innervation in the IML, MML and OML in our oldest cohort of animals (18 months old) using vGLUT2 labelling, a marker for presynaptic glutamatergic terminals [30]. We also quantified the total number of mossy cells in the dentate hilus. Since developmentally-born neurons are significantly more abundant in the dentate gyrus compared to ABNs, it is more likely that these quantifications correspond to developmentally-born neurons [31, 32]. We observed no differences in the dentate glutamatergic innervation across each sub-layer of the ML using vGLUT2 labelling (Figure S5), nor in the total number of mossy cells (Figure S6), regardless of the cognitive status at old age. Based on these observations, we propose that in our behavioral paradigm resilience to cognitive aging primarily relies on ABNs and their ability to maintain proper and functional integration into the neuronal network and circuitry during aging.

### Mitochondrial homeostasis in the proximal dendrites of ABNs is maintained in resilient rats

Given that PSD95 is expressed in spines, our previous results suggest that ABNs in the vulnerable population have a reduced number of spines in the IML. Such spine disruption could be linked to metabolic alterations. The mitochondrial network contributes to the formation, maintenance, elimination and functionality of spines [33, 34] and mitochondria depletion was shown to reduce spine density [35].

**Fig. 4:**
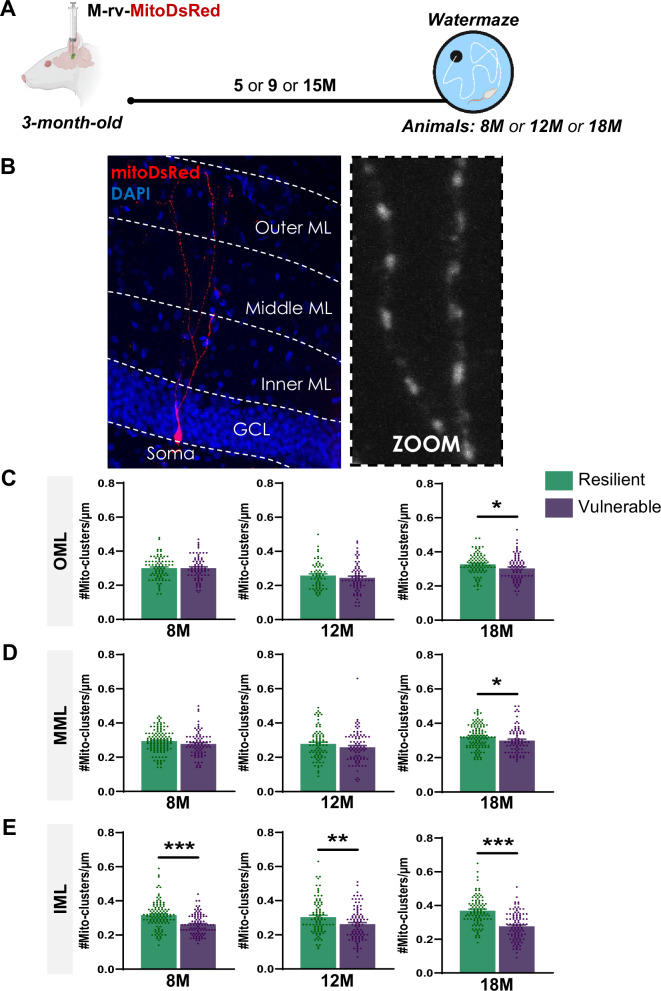
The mitochondrial homeostasis in the proximal dendrites of ABNs is maintained in resilient rats. **A** Schematic diagram of the experimental design. **B** Pictures of labelled ABN and illustration of the molecular layer (ML) division in three sub-layers; inner (IML), middle (MML) and outer (OML) molecular layer. **C** Resilience to cognitive aging is linked to the preservation of the mitochondrial density of the OML at 18-month-old (unpaired *t* test: *t*_*171*_ = 2.33, P ≤ 0.05) but not earlier at 8-month-old (unpaired *t* test: *t*_*154*_ = 0.01, P > 0.05) or 12-month-old (unpaired *t* test: *t*_*134*_ = 0.97, P > 0.05). **D** Resilience to cognitive aging is linked to the preservation of the mitochondrial density of the MML at 18-month-old (unpaired *t* test: *t*_*198*_ = 2.01, P ≤ 0.05) but not earlier at 8-month-old (unpaired *t* test: *t*_*203*_ = 1.81, P > 0.05) or 12-month-old (unpaired *t* test: *t*_*168*_ = 1.41, P > 0.05). **E** Resilience to cognitive aging is related to the preservation of the mitochondrial density of the IML at 8-month-old (unpaired *t* test: *t*_*208*_ = 5.53, P < 0.001), 12-month-old (unpaired *t* test: *t*_*183*_ = 2.91, P < 0.01) and 18-month-old (unpaired *t* test: *t*_*202*_ = 7.42, P < 0.001). Data are presented as mean ± S.E.M. from 5 extreme animals per group (a min of 3 neurons were analyzed per animal, with 8M-Res = 28 neurons and 8M-Vul = 25 neurons; 12M-Res = 20 neurons and 12M-Vul = 20 neurons; 18M-Res = 27 neurons and 18M-Vul = 27 neurons). Statistical significance *P ≤ 0.05, **P < 0.01, ***P < 0.001.

According to these observations, we followed the evolution of the mitochondrial network in ABNs with the use of a M-rv-MitoDsRed [19] (Fig. 4A), labelling their mitochondrial network. The quantification of the mitochondrial clusters density along the dendrites was performed in the IML, MML and OML as previously described (Fig. 4B). For all cognitively characterized aged-populations (Figure S1D), ABNs in vulnerable individuals exhibited a significant reduction in mitochondrial density specifically in the IML (Fig. 4E), aligning with the observed decrease in post-synaptic densities in the same layer. This mitochondrial deficit was initially restricted to the IML but extended to the OML and MML in old age (Fig. 4C, D), suggesting a progressive spread of mitochondrial dysfunction with aging. In contrast, resilient animals maintained mitochondrial density in the IML across all ages (Fig. 4E), indicating that the preservation of mitochondrial homeostasis in ABNs may be critical for maintaining synaptic integrity and supporting successful cognitive aging.

### Stimulation of ABNs restores memory retrieval in vulnerable animals

Taken together, the previous data indicate that the observed rarefaction of inputs and disruption of mitochondrial homeostasis could be attributed, at least in part, to a reduction in the functionality of ABNs. To by-pass these alterations, we artificially stimulated ABNs using an optogenetic approach according to a previously used protocol [36].

**Fig. 5:**
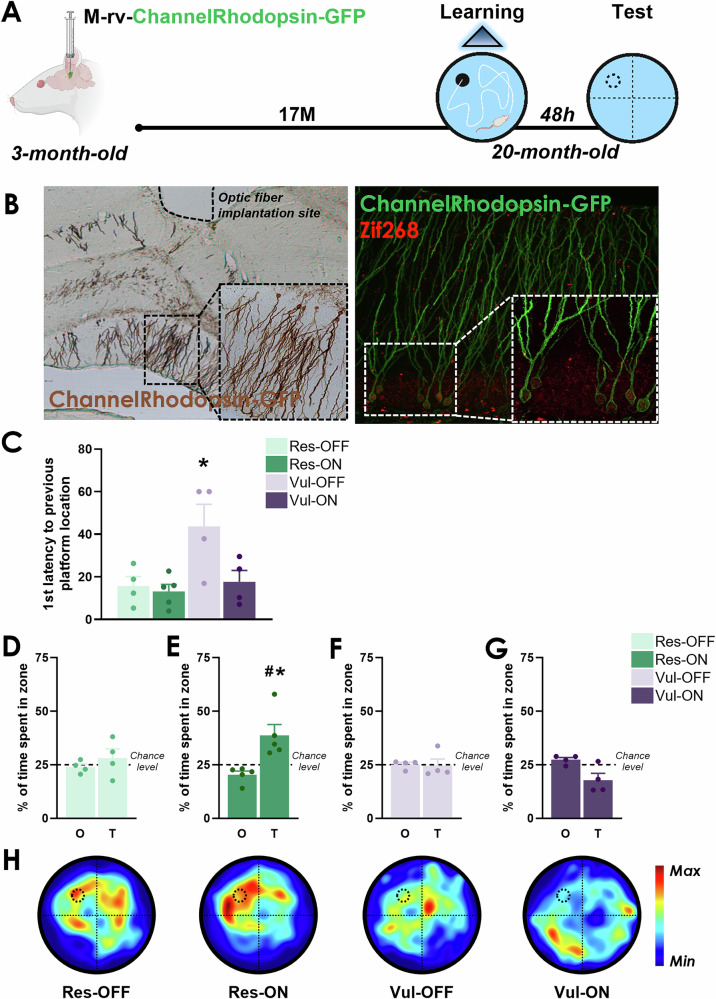
Stimulation of ABNs during learning restores memory retrieval in old vulnerable animals. O: Other quadrants, T: Target quadrant (**A**) Schematic diagram of the experimental design. **B** Picture of labelled ABNs (GFP^+)^ and optogenetically stimulated ABNs (GFP^+^-Zif268^+^). **C** Stimulation of ABNs in old stimulated vulnerable (Vul-ON) animals promoted the formation of a precise memory trace similar to resilient animals (Res-OFF and Res-ON) (One-way-ANOVA, F_(3, 13)_ = 5,23, P < 0,05). **D** Old non-stimulated resilient (Res-OFF) animals did not form a stable memory trace (paired *t* test: *t*_*3*_ = 0.72, P > 0.05). **E** Stimulation of ABNs in old stimulated resilient (Res-ON) animals promoted the formation of a strong memory trace (paired *t* test: *t*_*4*_ = 2.77, P ≤ 0.05 and one sample *t* test against ^#^chance level *t*_5_ = 3,23, P ≤ 0.05). **F** Old non-stimulated vulnerable (Vul-OFF) animals did not form a stable memory trace (paired *t* test: *t*_*3*_ = 0.12, P > 0.05). **G** Stimulation of ABNs did not restore the formation of a stable memory trace in Vul-ON animals (paired *t* test: *t*_*3*_ = 2.28, P > 0.05). **H** Heatmaps depicting the animals search location and occupancy during the memory test. Data are presented as mean ± S.E.M. Statistical significance *P ≤ 0.05, **P < 0.01, ***P < 0.001.

M-rv-ChannelRhodopsin-GFP was bilaterally injected in the dentate gyrus of 3-month-old rats. At old age (20-month-old, Fig. 5) or middle-age (12-month-old, Figure S7), animals underwent learning in the watermaze during which ABNs were optogenetically stimulated. Control animals followed the same procedure without light stimulation. Stimulation of ABNs did not improve the learning performances of animals (Figure S8). Two days after the end of learning, a probe test (during which the platform is removed) was conducted without stimulation to test for the memory of the task (Fig. 5A, B and Figure S7). We observed an improved memory of the former platform location in vulnerable animals stimulated during learning. More precisely, the stimulation of ABNs during learning promoted the formation of a more precise memory in vulnerable animals, as indicated by the decreased latency to reach the former platform position compared to the non-illuminated vulnerable group, reaching similar latency than for non-stimulated and stimulated resilient animals (Fig. 5C). Analysis of the percentage of time spent in the target quadrant (where the escape platform was previously located) revealed that the stimulation of ABNs had further enhanced the memory strength in resilient rats (Fig. 5D-G), as confirmed by the heatmaps analysis of the probe test (Fig. 5H). This indicates that optogenetic stimulation of ABNs in vulnerable rats was sufficient to restore memory retrieval to levels observed in non-stimulated resilient animals, but yet not enough to promote the formation of a strong and stable memory trace as it did in stimulated resilient animals. We observed similar results at middle-age (12-month-old – Figure S7), an age at which vulnerable animals already exhibit input loss and mitochondrial deficits. A postmortem confocal analysis using the immediate early gene Zif268 as a proxy of neuronal activity revealed that the stimulation levels of ABNS (M-rv-channelrhodopsin-GFP^+^ cells expressing Zif268) were similar at both ages between the two stimulated cognitive populations (Figure S9). Altogether, these results indicate that the stimulation of ABNs can restore memory retrieval in vulnerable animals and even further increase the stability and strength of memory in resilient animals.

## Discussion

Our results highlight the essential role of long-lived ABNs in successful cognitive aging. By using a novel strategy aiming at tagging ABNs generated during early adulthood, we followed their health and network integration throughout aging. We observed that ABNs in animals with reduced memory abilities progressively lose their glutamatergic innervation in their proximal dendrites, an effect maintained throughout life. This loss of inputs does not appear to be associated with the death or gross morphological deterioration of ABNs. However, we observed a similar reduction of the mitochondrial network density in the proximal dendrites of the ABNs. The reduced mitochondrial density may result from mitochondrial dysfunctions or deposition, which are calcium and activity-dependent [37]. Reduced local activity could generate local deposition of mitochondria and hinder their trafficking to the IML. Given that mitochondria represent essential hubs for synaptic functioning and spine maintenance and that mitochondria depletion was shown to reduce spine density [35], these data suggest a link between the loss of spines (using PSD95 as a proxy) and mitochondria alterations.

We propose that the loss of glutamatergic excitatory innervation, replicated in three independent cohorts of aged rats, may underlie the diminished responsiveness of ABNs observed during learning in vulnerable rats [16]. Glutamatergic inputs to the proximal dendrites in the IML originate from mossy cells and neurons in the SuM. While little is known about how these projections are affected in cognitive aging, existing studies have implicated both mossy cells [38–40] and SuM neurons [41, 42] in spatial memory retrieval. Their disruption may thus contribute to the cognitive deficits observed in vulnerable individuals. Interestingly, this disruption appears selective to the IML, raising the question of why ABN inputs in this layer are specifically affected. One possibility is that the origin of the dysfunction lies in the presynaptic neurons projecting to the IML, which may become impaired with aging, either through degeneration, altered firing, or loss of connectivity. Another hypothesis is that ABNs’ proximal dendrites may be more vulnerable to local metabolic stress or synaptic destabilization. Although we observed mitochondrial impairments in the IML of vulnerable rats, it remains unclear whether these represent a causal mechanism disrupting synaptic input, or a downstream marker of presynaptic degeneration. Future work should aim to dissect whether mitochondrial dysfunction is a driver or a consequence of synaptic loss in the IML. Nevertheless, our findings identify mossy cells and the SuM as key candidate contributors to resilience in cognitive aging, with functional alterations that may begin as early as middle age.

To date, only the connectivity of the MML and OML of the dentate gyrus had been addressed in the context of inter-individual differences in cognitive aging. Using electronic microcopy, the number of axo-spinous synapses was shown to be reduced in the MML of 24–28-month-old rats exhibiting memory deficits [43]. Along the same lines, the synaptophysin levels, a marker for pre-synaptic vesicles, indicated impaired connectivity in the MML and OML of vulnerable rats [44]. These results suggested that resilience to cognitive aging is linked to the preservation of inputs from the entorhinal cortex. While we did not observe differences in the post-synaptic density of MML and OML, we observed the emergence of alterations in the mitochondrial network of ABNs in these outer layers in 18-month-old vulnerable rats (Fig. 4C, D). As previously proposed, mitochondrial disruptions might lead to spine disruption and to the loss of inputs on the former spine. Given that the previous studies addressed the dentate connectivity in older rats aged 24–28-month-old [43, 44], it likely allowed them to identify alterations that we might have detected with our PSD95 tool at timepoints beyond 18-month-old in the MML and OML. Nevertheless, we cannot exclude that the tool used in our present study is more coarse-grained than electronic microscopy, suggesting we may have missed changes in entorhinal inputs. In addition, we cannot exclude the involvement of other inputs. For example, cholinergic inputs from the medial septum project onto dentate neurons and make asymmetric and presumably excitatory contacts on dendritic spines, chiefly in the IML [45], and are known to degenerate in vulnerable rats [46].

We propose that resilience to cognitive aging in our behavioral paradigm specifically relies on ABNs, rather than on dentate neuron generated during development. Indeed, developmentally-born dentate neurons are not recruited by spatial learning in either young adults [36, 47–49] or aged rats [16] that successfully acquire the task. Furthermore, they are not required for memory retrieval and reconsolidation either [47, 49]. To further support this hypothesis, we also observed no differences in the total glutamatergic innervation in the IML, MML, and OML using vGLUT2 labelling or in the total number of mossy cells in the dentate hilus in our oldest cohort between resilient and vulnerable animals (Figure S5 and Figure S6). Given that developmentally-born neurons are significantly more abundant in the dentate gyrus compared to ABNs, these quantifications likely correspond to developmentally-born neurons [31, 32]. Based on these observations, we propose that in our behavioral paradigm, resilience to cognitive aging primarily relies on ABNs and their ability to maintain proper and functional integration into the neuronal network during aging, especially with mossy cells and the SuM.

That said, we do not rule out the potential contribution of developmentally-born neurons to other aspects of behavior or memory processing. Learning in the watermaze is inherently complex, reflecting the multifaceted role of the hippocampus, which encodes both contextual and spatial information. Contextual cues may include a variety of stimuli: external visual cues, tactile sensations, vestibular and proprioceptive inputs, auditory information (though less critical for this task), as well as the animal’s internal states such as attention and motivation. The hippocampus also encodes multiple types of spatial information, including goal location, orientation, movement sequences, and both egocentric and allocentric spatial representations. Importantly, animals are trained over multiple trials across several days. During the initial phase, they explore the environment without prior knowledge of the platform’s location, allowing for the first encoding of spatial information. This is followed by consolidation and reconsolidation phases, which are progressively reinforced throughout training. It is therefore plausible that developmentally-born neuronal populations contribute to distinct behavioral or cognitive processes during the early phases of task acquisition, even if they are not directly involved during the latter, expert stages.

By stimulating long-lived ABNs, we successfully restored memory retrieval in both middle-aged and aged vulnerable rats, as evidenced by the improved latency to reach the former platform location, an effect reflecting enhanced memory precision. These results indicate that ABNs remain functionally viable in vulnerable animals and are capable of transmitting information for memory processing when activated. Their preserved output connectivity suggests that the primary barrier to their recruitment therefore lies in the rarefaction of afferent inputs. However, stimulation did not fully restore memory strength to the level observed in resilient stimulated animals. This suggests that additional factors—such as intact afferent inputs from mossy cells and/or the SuM, both preserved in resilient animals—may be essential for establishing a stronger and more durable memory trace. Alternatively, since only a relatively small population of ABNs born during young adulthood was targeted in our study, a stronger rescue may have been possible by stimulating a larger ABN population in vulnerable individuals.

Despite this limitation, the observed enhancement in memory precision after activating a small number of ABNs raises an important question: how can the selective recruitment of such a limited neuronal population exert such a strong influence on memory performance? Although the literature on long-lived ABNs remains sparse, several studies suggest that these neurons can exert a broad impact both within the hippocampus [50] and across broader brain networks [51], by modulating synaptic plasticity [52] and the temporal dynamics of network activity [53–56]. Efforts to fully answer this question are hindered by the limited understanding of the cellular substrates underlying spatial navigation in the watermaze [57]. Nevertheless, as previously discussed [47], it is reasonable to assume that long-lived ABNs fulfill all the criteria of engram cells [58]. While our study targeted only one cohort of mature ABNs, it is likely that other ABNs generated later in adulthood, along with other cell types—including mossy cells, interneurons, and glial cells—also contribute to the memory engram. In support of this heterogeneity, distinct neuronal ensembles have been shown to exist within a single contextual-fear memory engram [59]. Therefore, artificial activation of ABNs may serve as a gateway to recruiting broader engram components by reinforcing their network recruitment, ultimately promoting the formation of a more robust, brain-wide network that supports both the precision and strength of memory.

As mentioned, vulnerability to cognitive aging is characterized by reduced glutamatergic innervation and mitochondrial deficits specifically in the proximal dendrites of ABNs. These structural alterations may originate from dysregulation of the hypothalamic–pituitary–adrenal (HPA) axis, as animals with heightened stress reactivity and a hyperactive HPA axis are more prone to develop age-related memory impairments [12, 60, 61]. Moreover, both HPA axis activity and memory abilities have been associated to the rate of neurogenesis in older animals [12]. Notably, reducing glucocorticoid exposure starting in middle-age —rather than during the early postnatal development [62]— has been shown to enhance neurogenesis in old age and prevent the onset of age-related memory disorder [12]. Given that glucocorticoid exposure disturbs synaptic density, mitochondrial homeostasis and trafficking [63, 64], as well as the synaptic integration of ABNs at young and old age [65, 66], it is plausible that early-life HPA axis hyperactivity —and the resulting prolonged glucocorticoid exposure– contributes to the structural deficits observed in vulnerable individuals. Glucocorticoid hormones exert a complex influence on memory, following an inverted U-shaped dose–response curve [67]. Although corticosterone secretion elicited by training in the watermaze [68] gradually attenuates with repeated testing [69], the inability to enhance memory strength in vulnerable animals may stem from this hyperactive HPA axis [12, 60, 61]. In support of this, the balance between memory strength and specificity in fear conditioning has been shown to be modulated by exogenous administration of glucocorticoids [70]. These findings underscore the need to further investigate how hormonal dysregulation may impact engram formation during spatial learning.

In conclusion, our results indicate that brain resilience relies on the preservation of the integration of long-lived ABNs into the network and circuitry. Our findings reveal their potential as a valuable tool for rejuvenating memory abilities at old age. Future therapeutic approaches aimed at restoring memory functions should target long-lived ABNs, as they clearly still participate in memory processes, a fact often overlooked in the literature. However, it is crucial to address the alteration of inputs detected as early as middle-age and to aim at preventing it to achieve optimal therapeutic results in old age.

## Supplementary information

Supplemental material

## Data Availability

The data that support the findings of this study are available from the corresponding author upon reasonable request.
