# Supplementary material for: Long-lived adult-born hippocampal neurons promote successful cognitive aging
Source: Mol Psychiatry. 2025 Oct 1;31(1):217–30. doi: 10.1038/s41380-025-03286-5 (PMC12700793; doi:10.1038/s41380-025-03286-5)
Supplement: Supplementary file 1 — Supplemental material [file 41380_2025_3286_MOESM1_ESM.docx]

**Supplementary information for:**

**Long-lived adult-born hippocampal neurons promote successful cognitive aging**

**Authors:** Nicolas Blin^1^, Vanessa Charrier^1^, Fanny Farrugia^1^, Justine Palhol^2^, Antoine Presset^1^, Estelle Cartier^1^, Stephane Oliet^2^, Emilie Pacary^1^, Muriel Koehl^1^, Dieter Chichung Lie^3^, Nuria Masachs^1^, Djoher Nora Abrous^1*^

**Affiliations:**

^1^Univ. Bordeaux, INSERM, Magendie, U1215, Neurogenesis and Pathophysiology Group, F-3300 Bordeaux, France.

^2^Univ. Bordeaux, INSERM, Magendie, U1215, Glia-neuron interactions Group, F-3300 Bordeaux, France.

^3^Institute of Biochemistry, Emil Fischer Center, Friedrich-Alexander Universität Erlangen-Nürnberg, Erlangen, Germany.

**Corresponding author e-mail:**

e: nora.abrous@inserm.fr

tel: 33 (0)5 57 57 36 65

fax: 33 (0)5 57 57 36 69

Institut François Magendie

146 rue Léo Saignat

33077 Bordeaux Cedex – France

**Keywords:** Cognitive aging, resilience, inter-individual differences, dentate gyrus, adult neurogenesis, optogenetics, thymidine analogues, moloney murine leukemia virus.

#
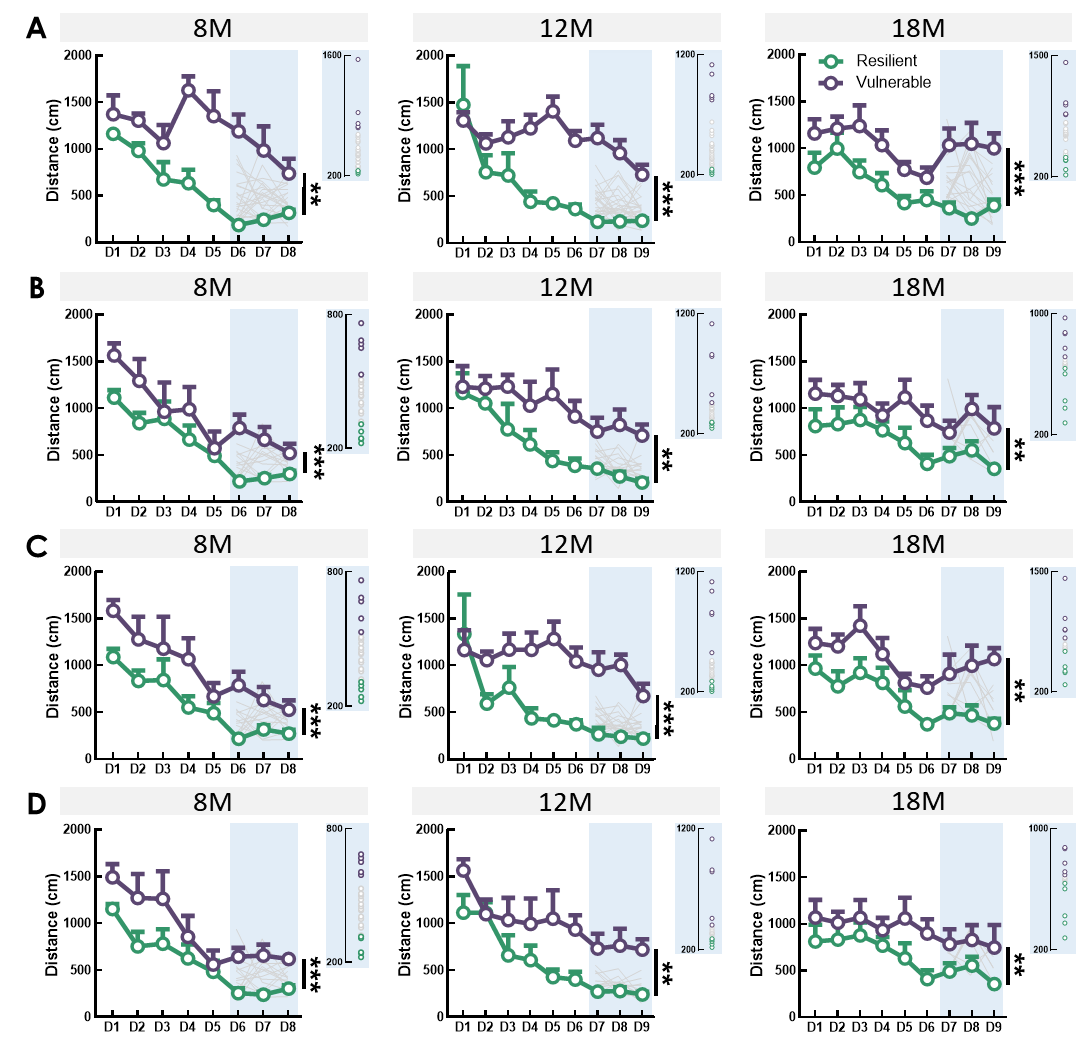
Supplementary figures S1 to S6

**Figure S1: Resilient animals show better learning abilities than vulnerable animals at each age.**

For each panel, the left graph shows the learning performance in the water maze of animals from the extreme resilient (green) and vulnerable (purple) populations across days of training, along with intermediate individuals (grey) shown for the last three days of training. The right graph displays inter-individual variability within the animal cohort subjected to spatial learning, averaged over the last three training days, including resilient (green), vulnerable (purple), and intermediate (grey) animals, used for the cognitive group selection (see Materials and Methods section for selection criteria).

(A) Cohorts used for the survival and senescence analyses with BrdU and SAβGal. Resilient animals show better learning performances at 8-month-old (RM-two-way ANOVA, F_1,8_=23.10, P < 0.01), 12-month-old (RM-two-way ANOVA, F_1,8_=140.8, P < 0.001) and 18-month-old (RM-two-way ANOVA, F_1,8_=40.43, P < 0.001).

(B) Cohorts used morphological analysis with M-rv-CAG-GFP. Resilient animals show better learning performances at 8-month-old (RM-two-way ANOVA, F_1,8_=99.18, P < 0.001), 12-month-old (RM-two-way ANOVA, F_1,8_=16.09, P < 0.01) and 18-month-old (RM-two-way ANOVA, F_1,8_=21.22, P < 0.01).

(C) Cohorts used for glutamatergic innervation analysis with M-rv-PSD95-GFP. Resilient animals show better learning performances at 8-month-old (RM-two-way ANOVA, F_1,8_=78.60, P < 0.001), 12-month-old (RM-two-way ANOVA, F_1,8_=38.50, P < 0.001) and 18-month-old (RM-two-way ANOVA, F_1,8_=18.01, P < 0.01).

(D) Cohorts used for mitochondrial network analysis with M-rv-MitoDsRed. Resilient animals show better learning performances at 8-month-old (RM-two-way ANOVA, F_1,8_=207.1, P < 0.001), 12-month-old (RM-two-way ANOVA, F_1,8_=12.52, P < 0.01) and 18-month-old (RM-two-way ANOVA, F_1,8_=16.89, P < 0.01).

Data are presented as mean ± S.E.M. from 5 extreme animals per group. Statistical significance *P ≤ 0.05, **P < 0.01, ***P < 0.001.


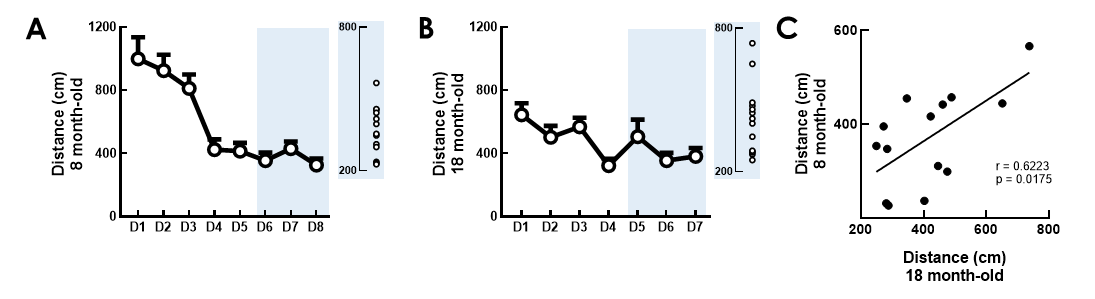


**Figure S2: The cognitive status of animals remains stable across aging.**

(A) Learning performance of a naive cohort of animals at 8 months of age. Data are presented as mean ± S.E.M.

(B) Learning performance of the same cohort at 18 months of age. Data are presented as mean ± S.E.M.

(C) Correlation between individual learning performance at 8 and 18-month-old, indicating stable cognitive status over time (Pearson correlation, P < 0.05).

**
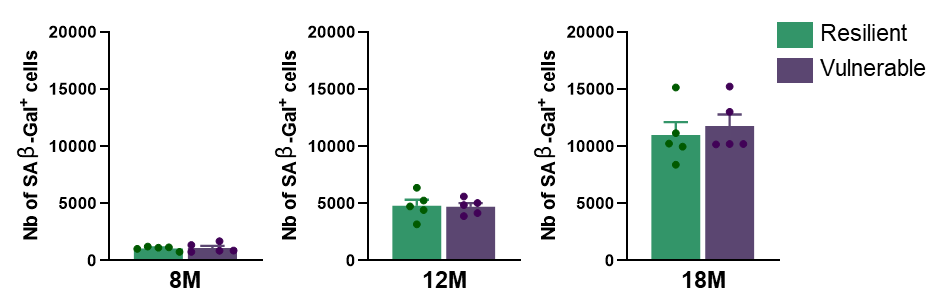
Figure S3: Cell senescence in the granule cell layer of the dentate gyrus do not account for successful cognitive aging.**

Resilient and vulnerable animals show similar number of senescent cells in the granule cell layer at 8-month-old (unpaired *t* test: *t_8_* = 0.28, P > 0.05), 12-month-old (unpaired *t* test: *t_8_* = 0.14, P > 0.05) and 18-month-old (unpaired *t* test: *t_8_* = 0.51, P > 0.05).

Data are presented as mean ± S.E.M. from 5 extreme animals per group. Statistical significance *P ≤ 0.05, **P < 0.01, ***P < 0.001.

**
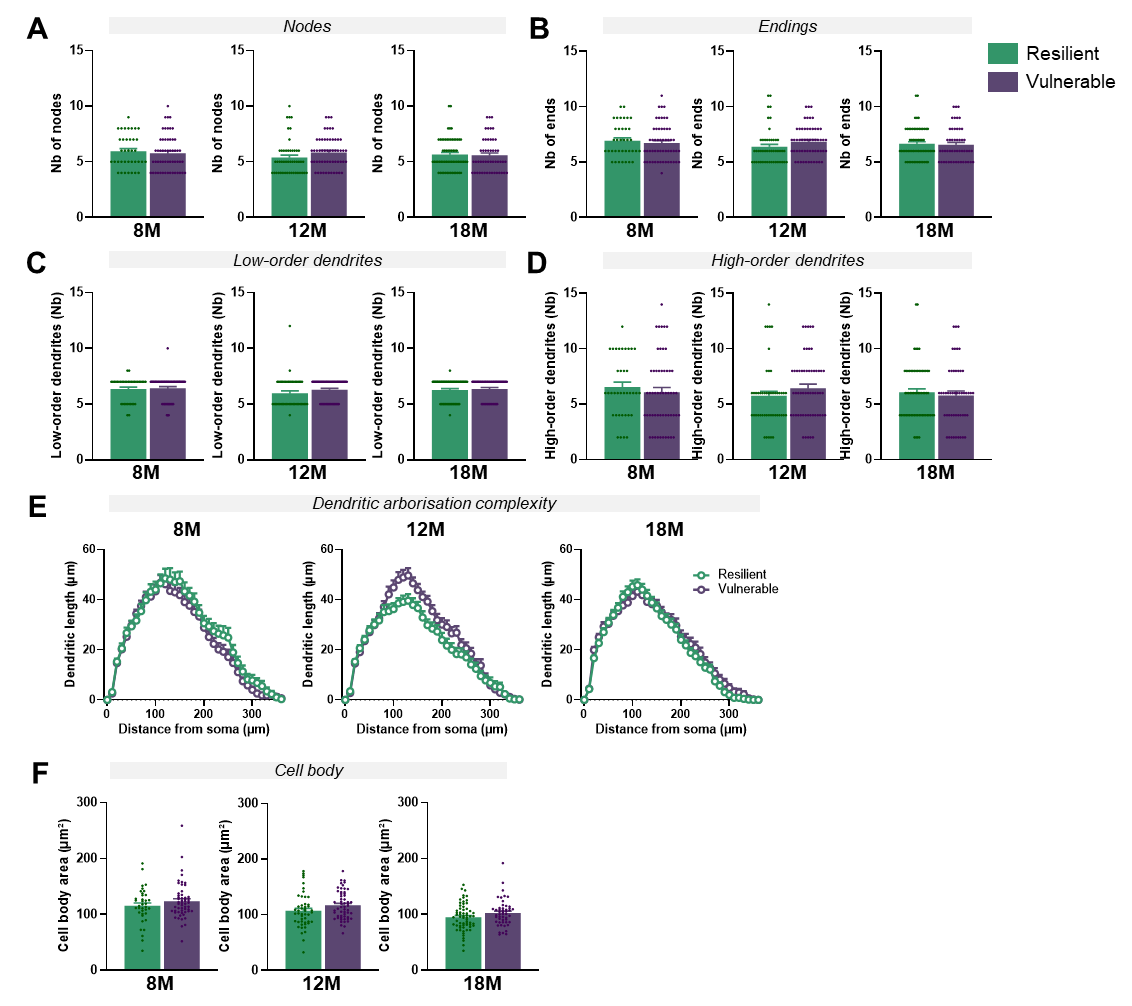
**

**Figure S4: The gross morphological architecture of ABNs is independent of the cognitive status of the rats.**

(A) ABNs in the two cognitive populations show similar number of nodes at 8-month-old (unpaired *t* test: *t_90_* = 0.55, P > 0.05), 12-month-old (unpaired *t* test: *t_102_* = 1.59, P > 0.05) and 18-month-old (unpaired *t* test: *t_111_* = 0.31, P > 0.05).

(B) ABNs have similar number of ends in resilient and vulnerable animals at 8-month-old (unpaired *t* test: *t_90_* = 0.64, P > 0.05), 12-month-old (unpaired *t* test: *t_102_* = 1.53, P > 0.05) and 18-month-old (unpaired *t* test: *t_111_* = 0.31, P > 0.05).

(C) ABNs in the two cognitive populations show similar low-order dendrites number at 8-month-old (unpaired *t* test: *t_90_* = 0.28, P > 0.05), 12-month-old (unpaired *t* test: *t_102_* = 1.33, P > 0.05) and 18-month-old (unpaired *t* test: *t_111_* = 0.48, P > 0.05).

(D) ABNs show similar high-order dendrites number at 8-month-old (unpaired *t* test: *t_90_* = 0.72, P > 0.05), 12-month-old (unpaired *t* test: *t_102_* = 1.22, P > 0.05) and 18-month-old (unpaired *t* test: *t_111_* = 0.02, P > 0.05).

(E) ABNs have similar dendritic complexity between the two cognitive populations at 8-month-old (RM-two-way ANOVA, F_36,3240_=0.68, P > 0.05) and 18-month-old (RM-two-way ANOVA, F_36,3996_=0.82, P > 0.05). At 12-month-old, a small difference could be detected between the resilient and vulnerable populations (RM-two-way ANOVA, F_36,3708_=2.59, P < 0.001).

(F) ABNs in the two cognitive populations show similar cell body area at 8-month-old (unpaired *t* test: *t_90_* = 1.22, P > 0.05), 12-month-old (unpaired *t* test: *t_102_* = 1.84, P > 0.05) and 18-month-old (unpaired *t* test: *t_111_* = 1.66, P > 0.05).

Data are presented as mean ± S.E.M. from 5 extreme animal per groups (a min of 4 neurons were traced per animal, with 8M-Res = 37 neurons and 8M-Vul = 55 neurons; 12M-Res = 48 neurons and 12M-Vul = 58 neurons; 18M-Res = 63 neurons and 18M-Vul = 50 neurons. Statistical significance *P ≤ 0.05, **P < 0.01, ***P < 0.001.

**
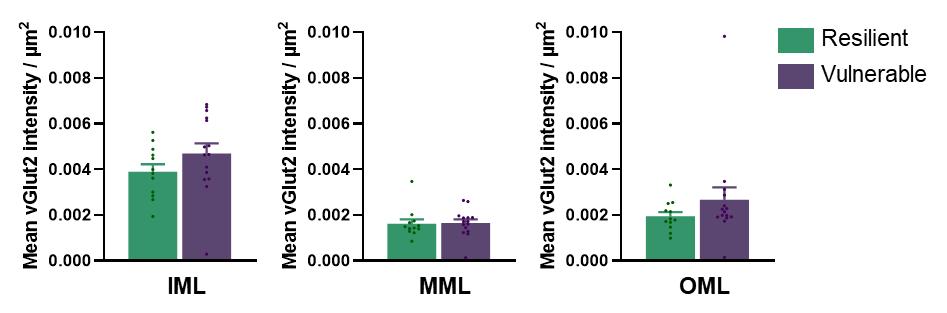
Figure S5: Similar glutamatergic innervation in the molecular layer of the dentate gyrus between resilient and vulnerable individuals at old age.**

Resilient and vulnerable animals show similar mean intensity for vGLUT2 labelling at 18-month-old in the IML (unpaired *t* test_25_: *t* = 1.38 P > 0.05), MML (unpaired *t* test: *t*_25_ = 0.09, P > 0.05) and OML (unpaired *t* test: *t_25_* = 1.12, P > 0.05).

Data are presented as mean ± S.E.M. from 5 extreme animals per group. Statistical significance *P ≤ 0.05, **P < 0.01, ***P < 0.001.

**
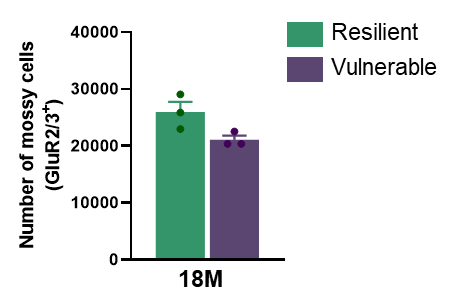
Figure S6: Similar number of mossy cells in the hilus of the dentate gyrus between resilient and vulnerable individuals at old age.**

Resilient and vulnerable animals show similar number of mossy cells expressing GluR2/3 at 18-month-old (unpaired *t* test: *t_4_* = 2.57, P > 0.05).

Data are presented as mean ± S.E.M. from 3 extreme animals per group. Statistical significance *P ≤ 0.05, **P < 0.01, ***P < 0.001.

**
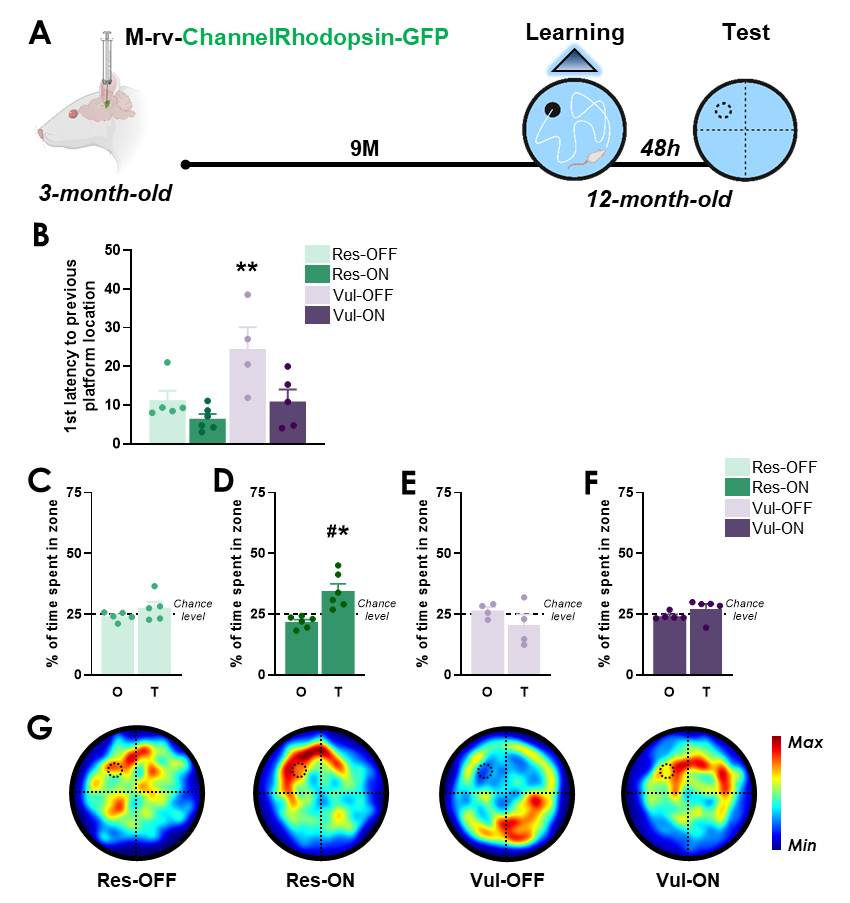
Figure S7: Stimulation of ABNs during learning restores memory retrieval in middle-aged vulnerable animals.**

O : Other quadrants, T: Target quadrant.

(A) Schematic diagram of the experimental design.

(B) Stimulation of ABNs in middle-age stimulated vulnerable (Vul-ON) animals promoted the formation of a precise memory trace similar to resilient animals (Res-OFF and Res-ON) (One-way-ANOVA, F_(3, 16)_ = 5,94, P < 0.01).

(C) Middle-age non-stimulated resilient (Res-OFF) did not form a stable memory trace (paired *t* test: *t_4_* = 1.08, P > 0.05).

(D) Stimulation of ABNs in middle-age stimulated resilient (Res-ON) animals promoted the formation of a strong memory trace (paired *t* test: *t_5_* = 3.23, P ≤ 0.05 and one sample *t* test against ^#^chance level *t*_5_ = 3,23, P < 0,001).

(E) Middle-age non-stimulated vulnerable (Vul-OFF) animals did not form a stable memory trace (paired *t* test: *t_3_* = 1.01, P > 0.05).

(F) Stimulation of ABNs did not restore the formation of a stable memory trace in Vul-ON animals (paired *t* test: *t_4_* = 1.15, P > 0.05).

(G) Heatmaps depicting the individuals search location and occupancy during the memory test.

Data are presented as mean ± S.E.M. Statistical significance *P ≤ 0.05, **P < 0.01, ***P < 0.001.


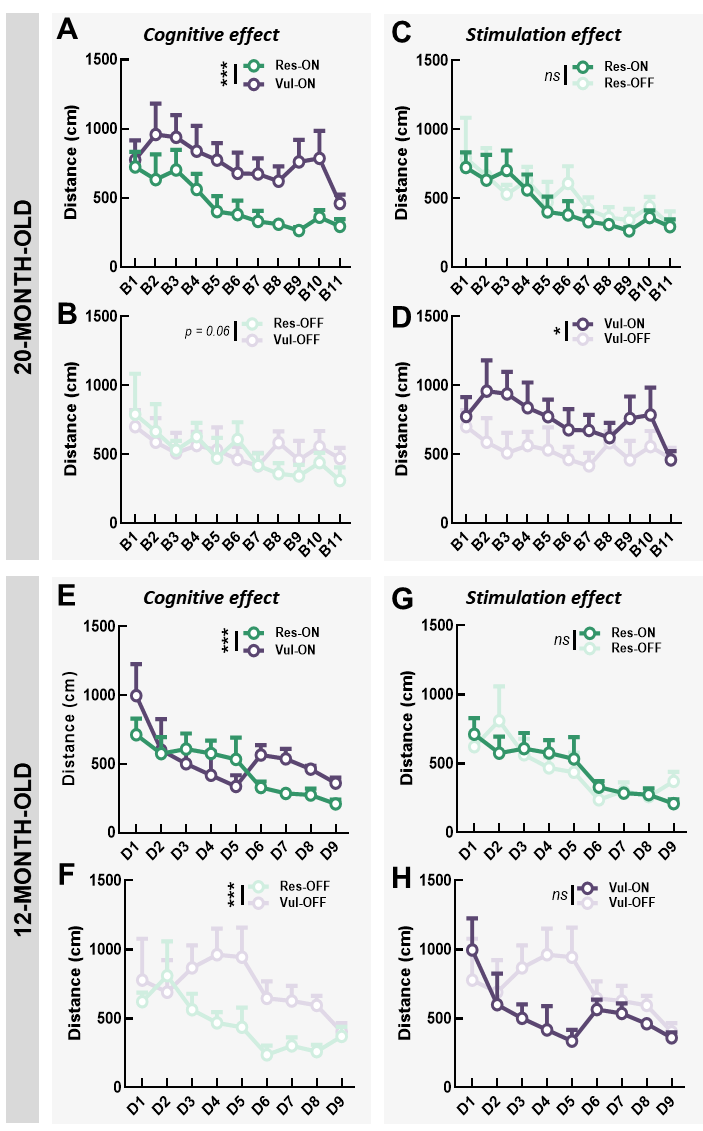


**Figure S8: Stimulation of ABNs during the learning phase has no effect on the performances independently of the cognitive status.**

(A) Old stimulated resilient (Res-ON) animals show better learning abilities than old stimulated vulnerable (Vul-ON) animals (RM-two-way ANOVA, three last days of learning, F_1,7_=29.31, P < 0.001).

(B) Old non-stimulated resilient (Res-OFF) animals show similar learning abilities than old non-stimulated vulnerable (Vul-OFF) animals (RM-two-way ANOVA, three last days of learning, F_1,6_=5.10, P > 0.05).

(C) Stimulation of ABNs did not improve the learning performance of old stimulated resilient (Res-ON) animals compared to old non-stimulated resilient (Res-OFF) animals (RM-two-way ANOVA, all learning days, F_1,7_=0.36, P > 0.05).

(D) Old non-stimulated vulnerable (Vul-OFF) animals show better learning performance than old stimulated vulnerable animals (Vul-ON) (RM-two-way ANOVA, all learning days, F_1,6_=11.78, P ≤ 0.05).

(E) Middle-age stimulated resilient (Res-ON) animals show better learning abilities than middle-age stimulated vulnerable (Vul-ON) animals (RM-two-way ANOVA, three last days of learning, F_1,9_=51.28, P < 0.001).

(F) Middle-age non-stimulated resilient (Res-OFF) animals show better learning abilities than midlle-age non-stimulated vulnerable (Vul-OFF) animals (RM-two-way ANOVA, three last days of learning, F_1,7_=76.48, P < 0.001).

(G) Stimulation of ABNs did not improve the learning performance of middle-age stimulated resilient (Res-ON) animals compared to middle-age non-stimulated resilient (Res-OFF) animals (RM-two-way ANOVA, all learning days, F_1,9_=0.11, P > 0.05).

(H) Stimulation of ABNs did not improve the learning performance of middle-age stimulated vulnerable (Vul-ON) animals compared to middle-age non-stimulated vulnerable (Vul-OFF) animals (RM-two-way ANOVA, all learning days, F_1,7_=3.38, P > 0.05).

Data are presented as mean ± S.E.M for: 5 animals, 20M-Res-ON: 5 animals; 20M-Vul-ON: 4 animals, 20M-Res-OFF: 4 animals, 20M-Vul-OFF: 4 animals, 12M-Res-ON: 6 animals, 12M-Vul-ON: 5 animals, 12M-Res-OFF: 5 animals and 12M-Vul-OFF: 4 animals. Statistical significance *P ≤ 0.05, **P < 0.01, ***P < 0.001.

**
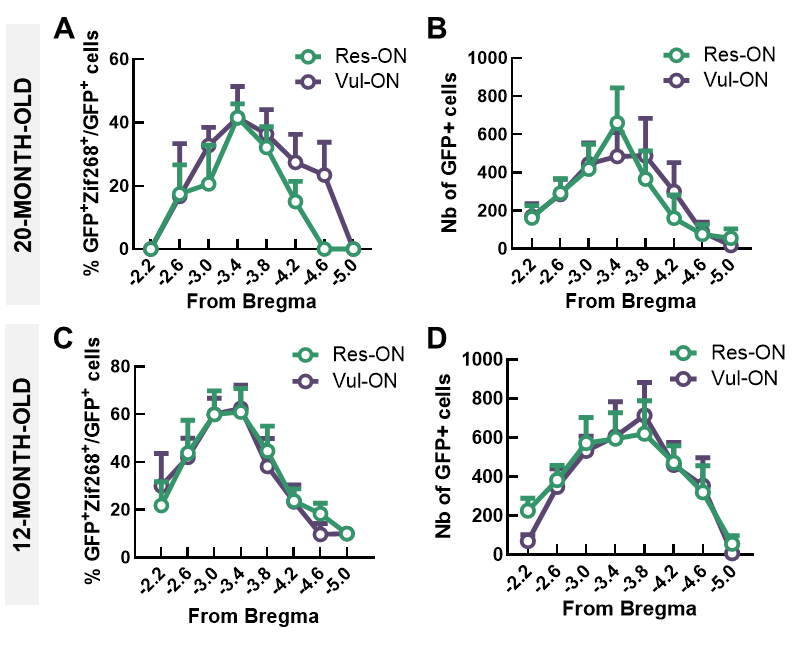
Figure S9: Similar levels of ABNs stimulation between resilient and vulnerable animals following light stimulation.**

(A) Similar percentage of ABNs stimulation between resilient and vulnerable animals at 20-month-old.

(B) Similar number of GFP labelled ABNs between stimulated resilient and vulnerable animals at 20-month-old.

(C) Similar percentage of ABNs stimulation between resilient and vulnerable animals at 12-month-old.

(D) Similar number of GFP labelled ABNs between stimulated resilient and vulnerable animals at 12-month-old.

Data are presented as mean ± S.E.M for 20M-Res-Light: 5 animals, 20M-Vul-Light: 4 animals,12M-Res-Light: 6 animals, 12M-Vul-Light: 5 animals. For the percentage of activation, data expressed as (Nb of GFP^+^-Zif268^+^ cells)/(Nb of GFP^+^- Zif268^−^ cells + Nb of GFP^+^- Zif268^+^ cells) × 100.
